# From Model Specification to Simulation of Biologically Constrained Networks of Spiking Neurons

**DOI:** 10.1007/s12021-013-9208-z

**Published:** 2013-11-20

**Authors:** Paul Richmond, Alex Cope, Kevin Gurney, David J. Allerton

**Affiliations:** 1 Department of Automatic Control and Systems Engineering, University of Sheffield, Sheffield, UK; 2 Department of Psychology, University of Sheffield, Sheffield, UK

**Keywords:** XML, Simulation, Translation, Interoperability, Spiking neurons, Neuronal networks

## Abstract

A declarative extensible markup language (SpineML) for describing the dynamics, network and experiments of large-scale spiking neural network simulations is described which builds upon the NineML standard. It utilises a level of abstraction which targets point neuron representation but addresses the limitations of existing tools by allowing arbitrary dynamics to be expressed. The use of XML promotes model sharing, is human readable and allows collaborative working. The syntax uses a high-level self explanatory format which allows straight forward code generation or translation of a model description to a native simulator format. This paper demonstrates the use of code generation in order to translate, simulate and reproduce the results of a benchmark model across a range of simulators. The flexibility of the SpineML syntax is highlighted by reproducing a pre-existing, biologically constrained model of a neural microcircuit (the striatum). The SpineML code is open source and is available at http://bimpa.group.shef.ac.uk/SpineML.

## Introduction

There is currently no clear consensus on the appropriate biological abstraction level for capturing the information processing aspects of the brain. Consequently there is a wide range of simulation tools and languages within the domain of computational neuroscience focusing on differing biological levels from networks of artificial neurons to molecular modelling of individual ion channels. In the context of specification and simulation of networks of spiking point neurons it is important that modelling tools provide a level of abstraction w is computationally efficient to simulate but while still allowing a sufficient degree of flexibility to describe a wide range of neuronal phenomena.

Conceptually and practically, it is also now recognised that it is desirable to separate the functions of a model *specification* and model *simulation*. The model specification should be easily human readable, unambiguous, and comprehensive, but also facilitate translation into executable code. Additionally, the model specification should be easily translated to multiple simulation platforms, both existing and new. Consider, for example, the microcircuit model described in (Humphries et al. 2009b) described by a mixture of hand written C and Matlab code. This is an anatomically accurate model of the striatum - the main input nucleus to the basal ganglia (a group of interconnected subcortical nuclei). The model uses 2-variable point neurons with physiologically realistic attributes such a dopaminergic modulation (Humphries et al. 2009a) and gap junctions (Humphries et al. 2009b). Translating this model into a higher level modelling description facilitates sharing, portability, repeatability and collaboration. Representation in a high-level format requires the flexibility to specify modified Izhikevich neuron body dynamics, enabling the behaviour resulting from differing ion currents to be captured. Similarly, to model inter-cell communication via gap junctions, support for neural components to communicate without using typical ‘synaptic’ connectivity is also essential.

An ideal modelling format to describe biologically constrained models would be simulator independent. Aside from specification clarity, simulator independent formats allow a developer to cross-check models through execution on a range of simulator engines (assuming simulator support is available). A number of simulator independent tools are available for describing a neural network models at various abstraction levels (Davison et al. 2009; Gleeson et al. 2010; Raikov et al. 2011). For the modelling requirements of the striatum, NineML (Raikov et al. 2011; Gorchetchnikov et al. 2011) was found to be the most suitable format. NineML was initiated by the INCFs Multiscale Modelling Program to address the limitations of both PyNN and NeuroML v.1. More specifically, it bridges the gap between the fixed library of standard neuron types in PyNN and the focus of conductance-based compartmental cell models in NeuroML by allowing neurons with arbitrary dynamics and networks with arbitrary connectivity to be expressed in a declarative simulator independent format. A library ‘libNineML’ exists for loading, saving and manipulating the NineML abstraction layer components as well as providing some translation of components to common simulators (NEST, Neuron and Brian). Unfortunately the current NineML format and simulator integration is incomplete, making it difficult to simulate complete network models described entirely in NineML.

**Fig. 1:**
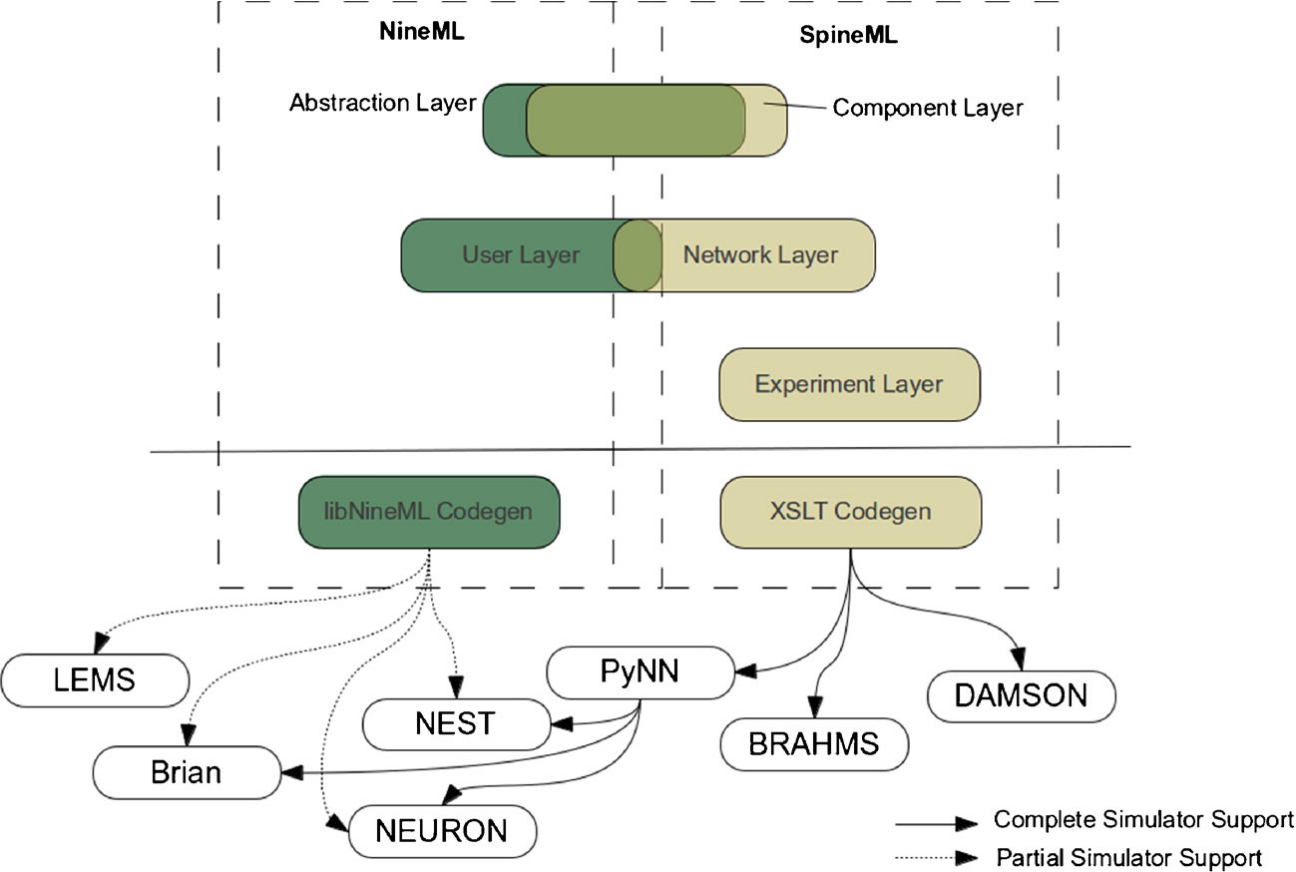
An incremental addition to the NineML model specification format. The SpineML syntax is a proposed extension to the NineML modelling format which provides a complete syntax for describing models of spiking point neuron models with varying biological complexity. In contrast with libNineML which provides code generation for only the component layer, the completeness of the SpineML syntax allows full simulator support for all three layers of components (neurons, synapses, etc.), networks and experiments

This paper builds upon NineML, utilising its layered inspired design (Raikov and De Schutter 2012) to propose an incremental extension to the existing NineML syntax. In particular a new network and new experimental description layer have been designed, both for maximising the flexibility of models which can be described (while retaining a point neuron focus) and providing a route for simple code generation of complete network model descriptions. The syntax proposed is given the name ‘SpineML’ (Spiking Neural Mark-up Language). Actual integration with the NineML core will be considered through INCF task force meetings. Figure 1 clarifies the relationship between NineML and the SpineML format. A three layer modelling approach is used to specify: components (e.g. Neurons, synapses, etc.), a network of connected components instances with individualised modelling parameters (e.g. differing starting membrane potentials) and an experimental layer describing the specification of a simulation such as runtime conditions, inputs and outputs.

This paper first outlines the design goals of the SpineML object format and toolchain before introducing examples of the syntax. Results from a benchmark model are presented in addition to results from reproducing a biologically constrained model of the striatum. The flexibility and the modelling syntax, code generation details and a description of the supported simulators are then presented. The paper concludes by discussing the advantages of the SpineML format in comparison with alternative notations.

## Design Goals

The primary design goal for extending the NineML syntax is to provide a simulator independent XML format to specify dynamics, network structure and experimental design of large-scale spiking neural network models. The SpineML format is based on a simulator independent XML format which facilitates a work flow promoting the use of independent content creation and simulation tools. The aims of the format can be summarised as:

1. To support the declarative specification of a wide range of (neuronal) dynamics and network connectivity.
2. To improve the ease and speed of model design and implementation.
3. To improve the understanding of model definitions through a clear descriptive format.
4. To facilitate and promote the sharing of models.

In order to meet these aims the following design goals are proposed;

### A separation of implementation from model description

minimises implementation error by reducing the modelling task to that of simply describing the biological system. This ensures models can be easily reproduced from mathematical and diagrammatic descriptions which are common in journal publications. This design goal is highlighted as the main benefit of the NineML format. SpineML embraces this design goal by following the same layered specification which allows modular components describing neural dynamics to be expressed as differential equations rather than as solutions (i.e. implementations of equations using a given numerical solver) and by ensuring experimental details of a simulation (i.e. the numerical solver, duration, inputs and outputs and parameter changes) are specified separately from other aspects of the model description.

### A platform independent model description format

promotes collaborative working and meets the aim of facilitating the sharing of model data. Reducing external software dependencies allows model development to take place without access to simulation tools. SpineML uses a declarative XML syntax to describe all aspects of models and simulation experiments. The use of XML ensures models are represented in a stand alone plain text form achieving the maximum level of independence from external tools e.g. simulators or graphical modelling tools.

### A self explanatory syntax

ensures that models can be easily understood and interpreted without the addition of either ambiguous plain text descriptions or assumed knowledge of procedural programming languages. To achieve this goal, SpineML uses a high-level and highly structured model representation with an expressive syntax ensuring that the aims of improving model clarity and ease of creation are fulfilled.

### An unambiguous model description

requires a succinct form with a guarantee of syntactical correctness. SpineML ensures models follow a structured and syntactically valid format through the use of a declarative syntax governed by XML Schemas. A well structured syntax is essential for automated code generation as it ensures that simulation code can be generated which is free from errors. Most importantly, an unambiguous format ensures that independent tools can be used to create or simulate models. Finally schema integration within common XML editors (e.g. Eclipse, Visual Studio) encourages the use of content assistance and auto completion aiding the speed and ease of model creation.

### Allowing a wide range of biological phenomena to be represented

requires that model designers can go beyond a limited (library) set of pre-selected neuron and synapse implementations by providing equations to describe behaviour. Furthermore, model designers should not be constrained by the functional limitations of any particular simulator which would require hand-written code (which is considered the least portable and understandable form of representation). SpineML allows the description of such models by providing a clear separation between ‘components’ which describe behavioural (i.e. neurons and synapses) and the network which describes how components are connected.

### Good integration with existing simulators

should be demonstrated. It is inevitable that not all simulators will be able to provide support for all aspects of any proposed specification, particularly with respect to network connectivity. It is therefore proposed that there should be an extendible network description format for the SpineML specification. Primarily the network layer should consist of a ‘high-level’ format to describe populations and projections, concepts supported by the majority of point neuron simulators and a ‘low-level’ network format extension that allows communication other than through chemical synapses between components (e.g. gap junctions and neuromodulators) for a smaller subset of simulators which support this functionality.

## Object Model and Examples

**Fig. 2:**
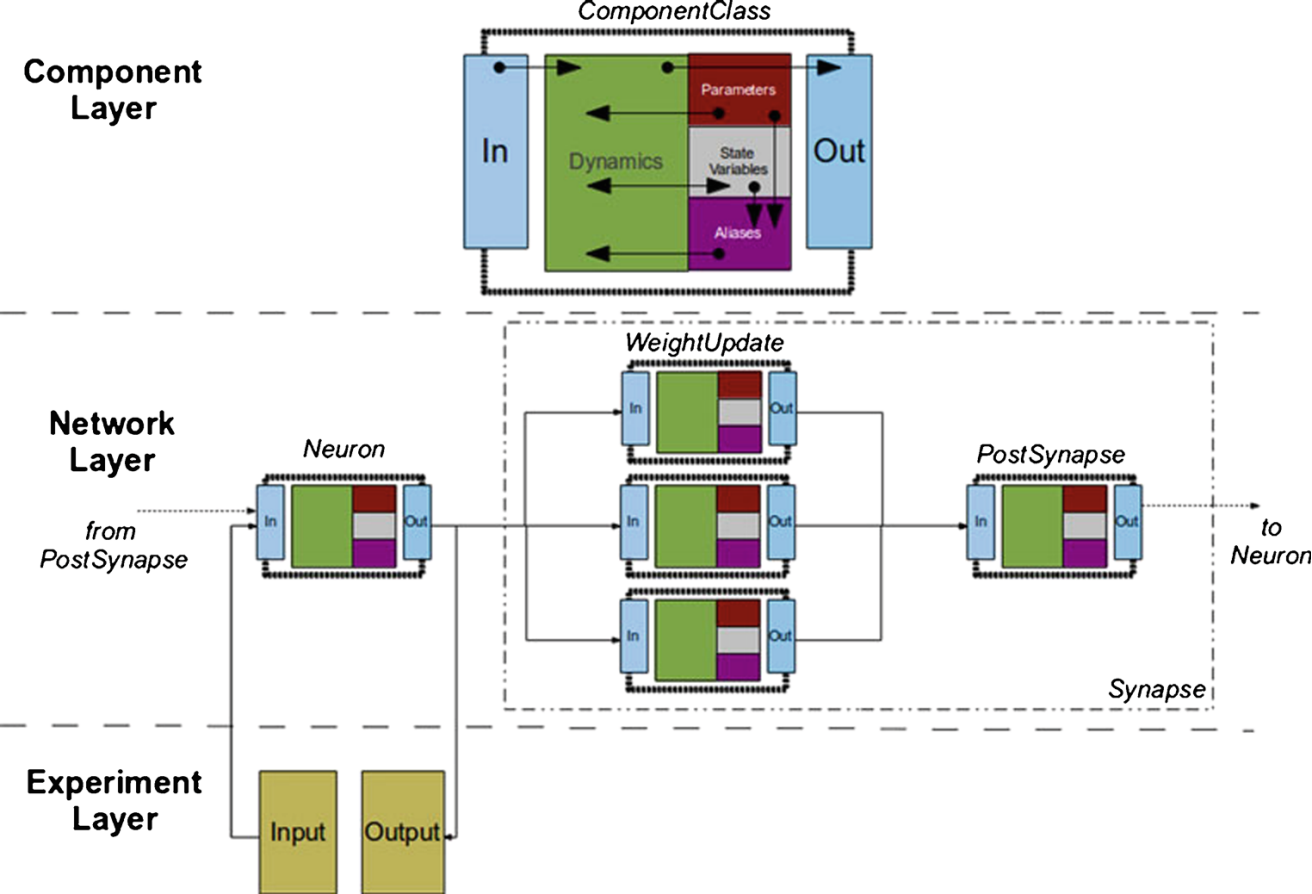
The modular dynamics within the three layers of SpineML. The figure shows the modular dynamical structure of the three layers of SpineML specification. A ComponentClass described within the component layer defines the dynamical behaviour of modular functional objects within a simulation (e.g. a neuron, synapse or neuromodulator). The dynamics of a ComponentClass manipulate state variables and emit **out**puts by evaluating sets of differential equations using only read-only parameters, **in**puts and aliases (an expression consisting of a mixture of parameters and state variables). The **In**put and **Out**put ports define an interface allowing a component instance (a component with a unique set of parameters and state variables) to be connected to other instances within the network layer. The figure shows the connectivity of a Neuron and Synapse (Composed of a set of WeightUpdates and a single PostSynapse). The experiment layer defines any additional inputs to the network of connected component instances, e.g. spike sources or current injections

The SpineML format is composed of three distinct layers of object model specification. This layered approach to modelling is inspired by the design goals of: separation of implementation from model descriptions and being able to express a wide range of dynamics. The three modelling layers within SpineML consist of: a ‘Component’ layer (with equivalent semantics to the NineML abstraction layer specification) for describing neuronal dynamics, a ‘Network’ layer (derived from the NineML user layer) describing a network of connected component instances and an ‘Experiment’ layer describing a series of simulations including inputs and outputs which form an ‘experiment’. Figure 2 illustrates the overall dynamics and modular connectivity of components for the three layers.

The following three sections of this paper describe each of the layers in detail using an example of a network of inhibitory and excitatory neurons (Vogels and Abbott 2005) to highlight the XML modelling syntax. The example has been selected because it has been subjected to extensive benchmarking (Brette et al. 2007) for a range of simulators.

### The Component Layer

Cellular level ‘ComponentClass’ definitions provide the building blocks of neural dynamics within a complete network model description. The ‘component’ level syntax of SpineML is directly derived from the NineML ‘abstraction’ layer which shares a number of features and common goals with LEMS^1^ and NeuroML 2.0.^2^ A complete object model for this layer is described on the NineML website and a complete Schema for SpineML is available on the SpineML website. The SpineML ‘component’ level and NineML ‘abstraction’ level differ only with respect to the syntax describing ports (shown in the subsequent examples) and that within SpineML dimensionality and unit are specified as a single SI attribute.

**Fig. 3:**
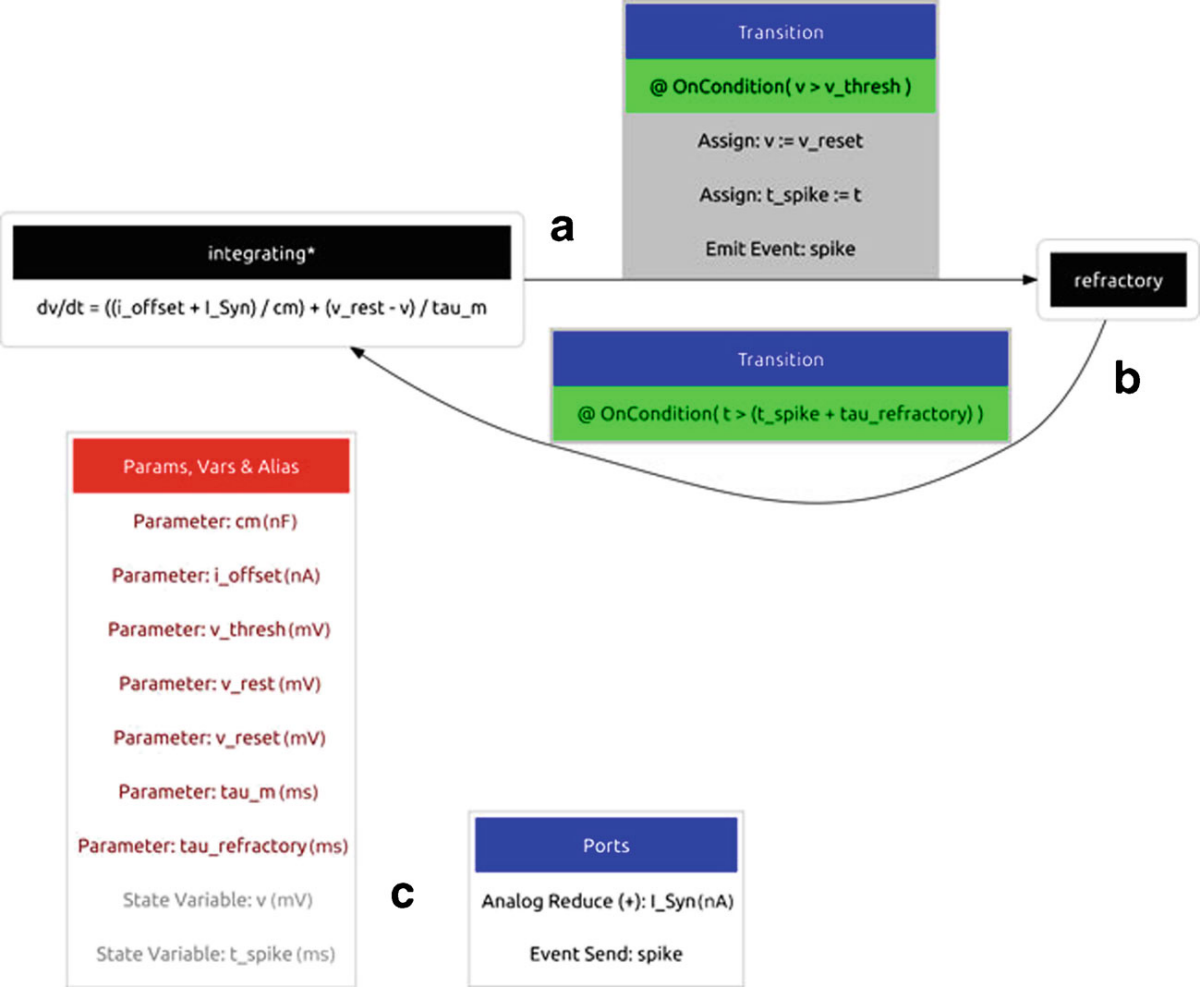
Visual representation of a Leaky Integrate and Fire (LIF) neuron body component. The component transitions between two Regimes (*black*) according to conditions which test internal state variables of the model. The refractory regime **b** has no behaviour but persists until the simulation time *t* exceeds the refractory period *tau_refractory*. The ‘integrating’ regime **a** is the initial regime, indicated by the asterisk symbol (*). All Parameters and State Variables referenced within any time derivatives or conditions are shown within the disconnected red box. A single analogue reduce port *I_Syn* which sums synaptic current inputs and an event output port *spike* are also shown (in the disconnected *blue box*) for completeness

A visual representation of the dynamics of an example component is shown in Fig. 3 which is visualised using the Graphviz library (Gansner et al. 1993). The component describes a standard Leaky Integrate and Fire (LIF) neuron body with refractory period. Graphical representation of this form gives a clear overview of the component demonstrating how state-like ‘regimes’ change the underlying dynamics in response to events and changing conditions during simulation. The SpineML definition of the LIF neuron body consists of two independent regimes of behaviour which define the dynamics of the model in differing states. Within a regime, a time derivative indicates a differential equation governing the progression of a named state variable. The following fragment of XML is a definition of the ‘integrating’ regime labelled A in Fig. 3. Line 4 defines how the voltage *v* changes over time. The regime has a transition (to a regime named ‘refractory’) triggered (line 7) when the voltage reaches a threshold level which instantaneously resets the voltage (line 8), emits a spike event (line 14) and also records the time of the spike emission (line 11). For clarity the symbol > is replaced with XML safe representations &*gt*; (line 16). Transitions are also able to assign state variables explicitly or emit events or impulses (events with a value). 
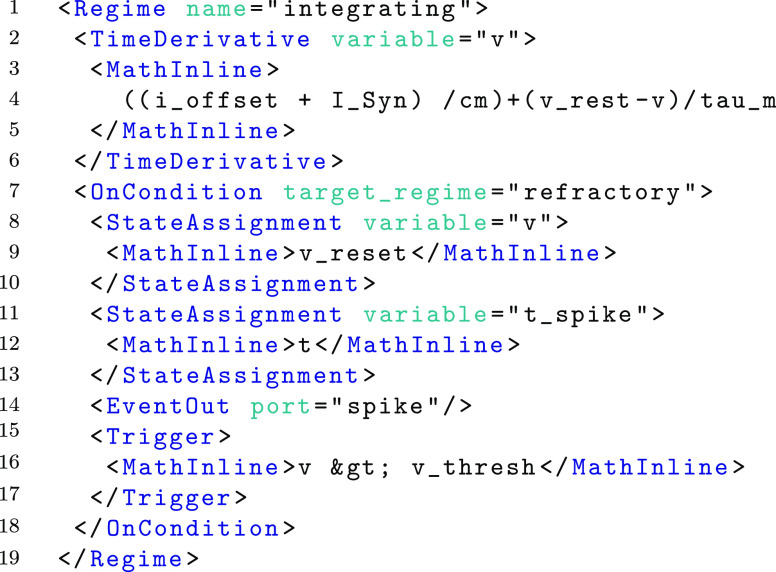

A subsequent regime ‘refractory’, labelled within Fig. 3 as B is described below. A Trigger condition (line 3) causes the component to transition into the ‘integrating’ regime after a *tau*_*refractory* period of time. 
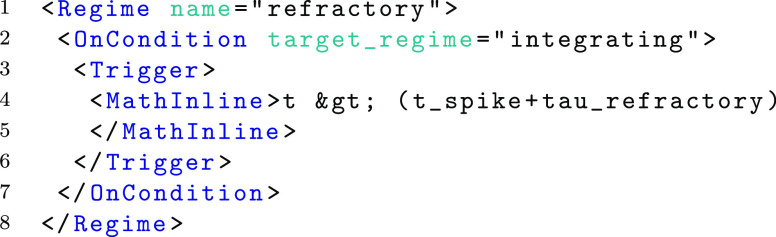


Aside from regimes of a component, state variables form the final specification of the dynamics. The following example shows the complete LIF component class (with the two regimes omitted). Two state variables *v* and *t*_*spike* are shown on lines 15 and 16 respectively. Parameters are non-changing variables of the model which are referenced by time derivatives, state assignments and triggers (i.e. wherever a MathInline element is used). As with state variables, parameters have both a name and a dimension consisting of an SI unit. The SI unit can be converted into a separate dimension and unit using a simple lookup within simulators and modelling tools to ensure dimensional consistency. 
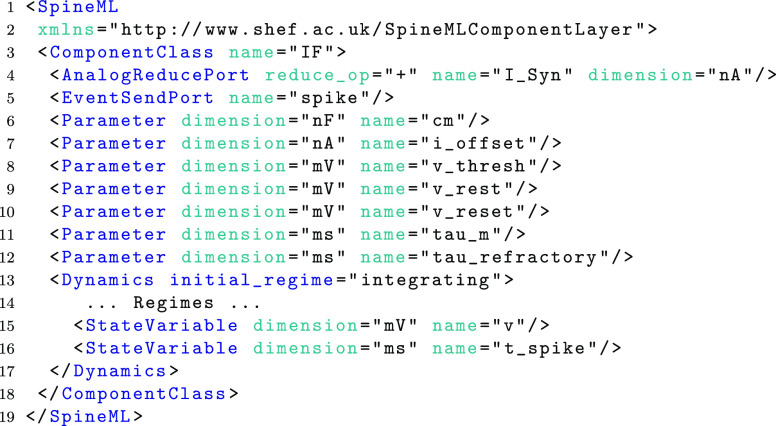


**Fig. 4:**
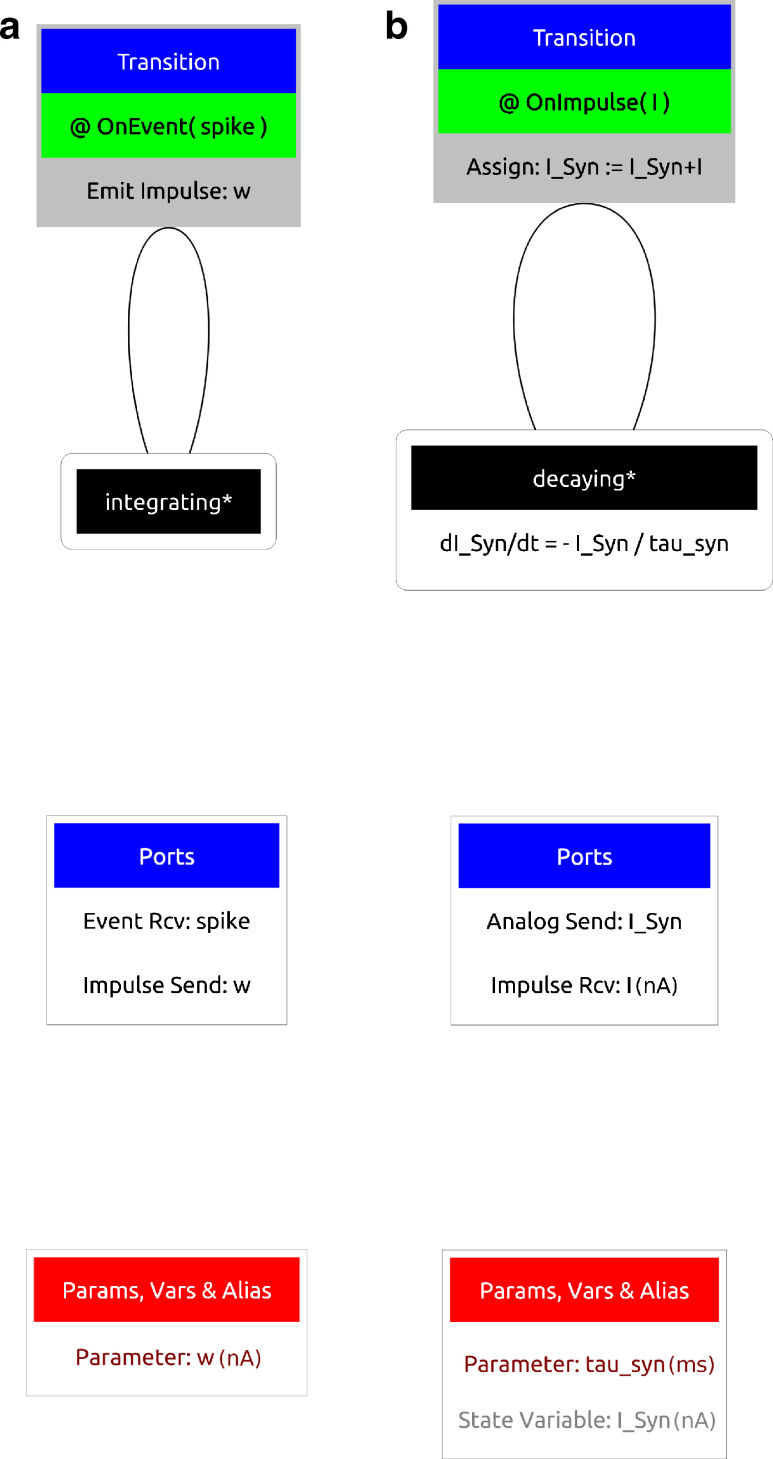
Diagrammatic representation of a synapse consisting of a fixed-weight update and post-synapse component. The figure shows a fixed-weight update component **a** which has a single event-based transition causing the emission of an impulse of the synaptic weight. **b** Shows a exponentially decaying post-synaptic current which decays according to the shown time derivative. On the receipt of an impulse the post-synaptic current (I_Syn) is increased by the impulse value

As shown in Fig. 2, ports are used within the ‘component’ layer as *hooks* to provide communication channels between components. Analogue ports send and receive values at each simulated time step and have a special case, a reduce port, which allows values from multiple components to be reduced (via a reduce operator) to a single value. The LIF neuron demonstrates an analogue (input) reduction port (line 4) by summing an input *I*_*Syn* representing synaptic current and an event send (output) port (line 5). Event and impulse (and event with a value) receive ports can be used to trigger transitions between regimes; as with conditional transitions, an OnEvent or OnImpulse can trigger state assignments or event and impulse outputs. Event ports and impulse ports are used within the component definitions of a fixed-weight update and an exponentially decaying post-synaptic current, shown in Fig. 4. The SpineML code for each component is available on the SpineML website.

In addition to parameters, an alias definition can also be used as a mechanism to group mathematical expression which refer to parameters, state variables and other alias definitions. For example an alias *refractory*_*end* could be defined as follows; 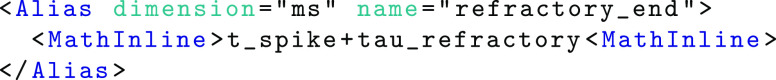


This new alias can then be used within the trigger expression of the condition linking the refractory regime to the integrating regime as follows; 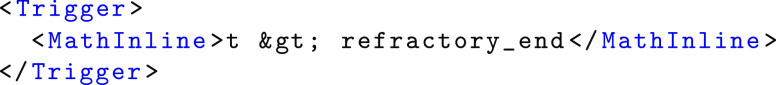


### The Network Layer

**Fig. 5:**
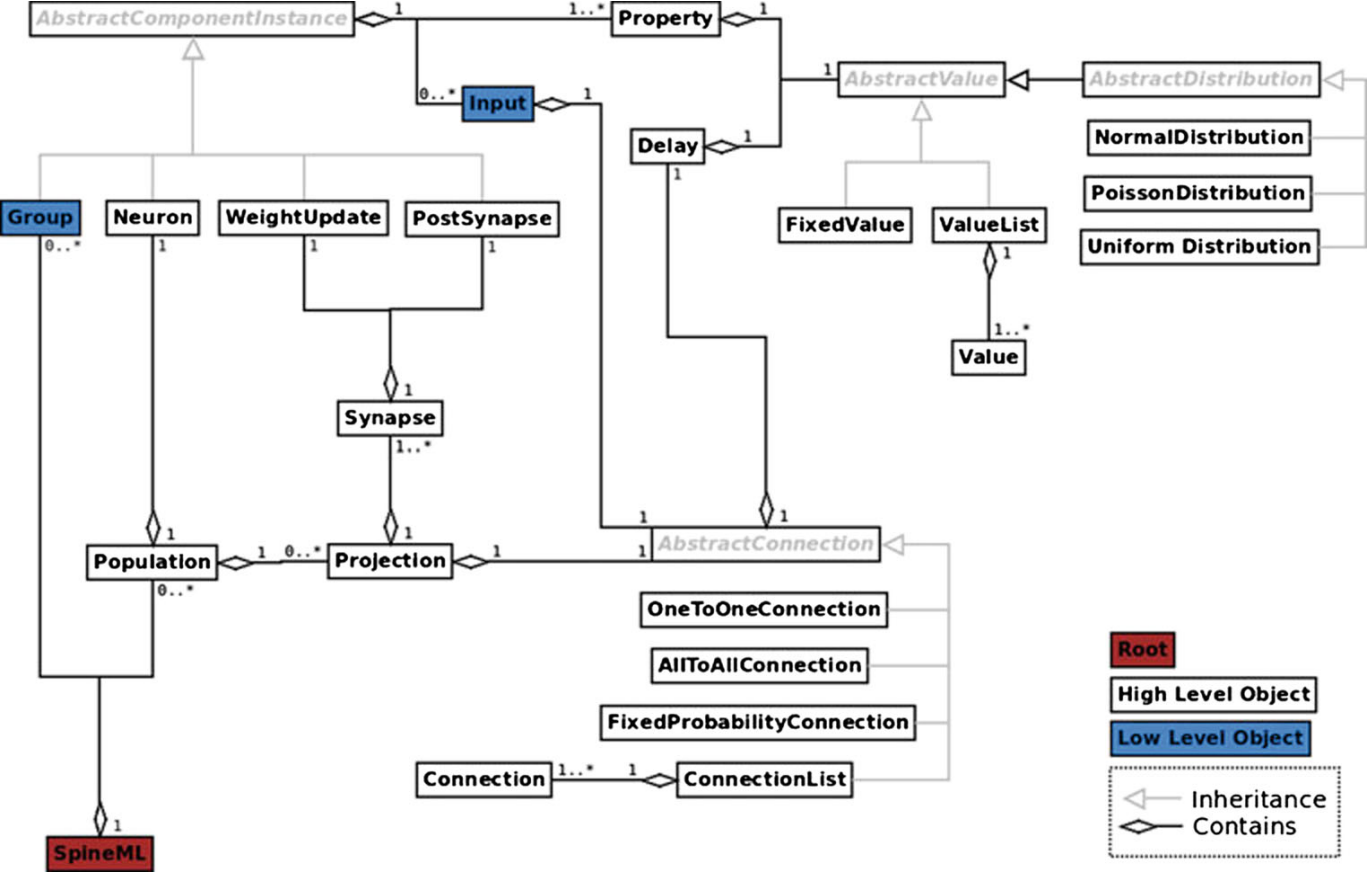
An object model for describing networks of connected components. The figure shows the object model of the SpineML Network Level syntax. Each box represents a high (*white*) or low (*blue*) level object corresponding to an XML element within the SpineML description format. The low level format contains only Input and Group allowing the definition of sets of component instances and connections which are able to communicate through mechanisms other than chemical synapses. Relationships between objects are indicated by ownership. i.e. a single a Population contains (or owns) a single Neuron and a single Population contains zero or more Projections. Abstract objects and the Inheritance relationship are shown in grey i.e. a Neuron, WeightUpdate, PostSynapse and Group all inherit AbstractComponentInstance which instantiates a component layer model description. A single SpineML object forms the root of the network layer

Following the specification of a set of ‘components’, the ‘network’ level description allows instances of components to be connected via ports using terminology familiar to neural modellers, e.g. populations and projections. The complete object model of the network layer is shown in Fig. 5; the SpineML website contains additional detailed element descriptions. The core object of the network layer is an AbstractComponentInstance, where each of the subclasses which inherit this object (i.e. Neuron, WeightUpdate, PostSynapse and Group) represents a set of instantiated components with unique property values. The high-level syntax imposes a building block structure using Neurons, WeightUpdates and PostSynapse primitives to form a hierarchy. More specifically, a Population can contain one or more Projections (to a named target population). Each Projection can contain one or more Synapses which have a unique connectivity pattern and sets of instantiated *WeightUpdate* and *PostSynapse* component models. Having a neuron clearly separated from the post-synaptic response allows multiple synaptic models (each with differing post-synaptic responses) to be projected to a neuron population. Separation of the model components in this way also allows for maximum flexibility, promoting re-usability of components, facilitating fast model design and improved model clarity. For example a fixed-weight synapse can be changed easily to a learning synapse without changing the post-synaptic response.

Continuing from the previous LIF component model, the following example demonstrates the syntax to describe an Excitatory population of LIF neurons. Each of the 3200 neurons within the population follow the model dynamics of the LIF component model which is referred to within an XML file ‘Integrate_and_fire.xml’ located by the *url* attribute. 
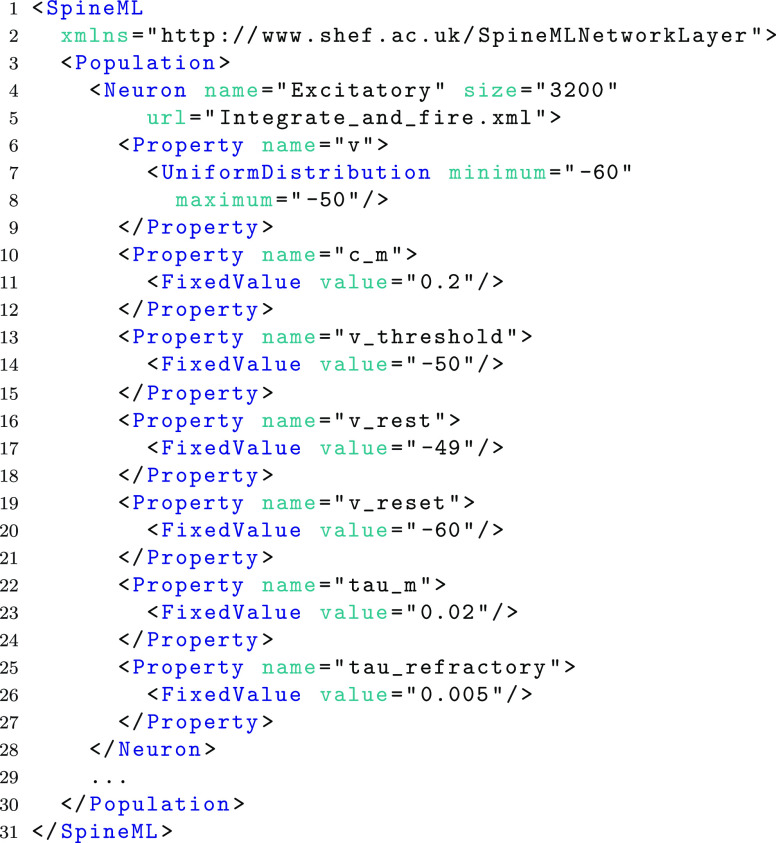


Within the neuron element (line 4) any number of properties may be defined. The property attribute *name* refers to either a parameter or state variable name from the component definition with values indicating the initial value prior to simulation of the component. Units and dimensionality are inherited from the component description. Parameters or state variables which do not set property values in the network layer (e.g. *i*_*offset* and *t*_*spike*) are assumed to have a default value of 0. Property values can be set using a fixed value for all instances (shown above using the *FixedValue* element), uniform, normal and Poisson distributions as well as explicit property lists. For example, as an alternative to using a normal distribution to specify the initial values of the neuron body voltage *v*, a value list can be used as shown in the following example. An attribute *index* is required to allow the specification of out of order or partial sets of explicit values. 
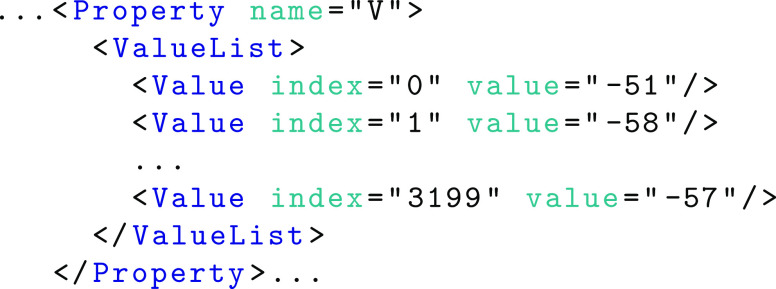


Using the high-level network syntax, a fixed probability synaptic connectivity (described using a *FixedProbabilityConnection* element) between the above ‘Excitatory’ population and an ‘Inhibitory’ destination population (described using the same syntax) can be expressed as is shown in the following example. Figure 5 shows the additional connectivity patterns. For further detail, readers are directed to the network layer documentation on the SpineML website. 
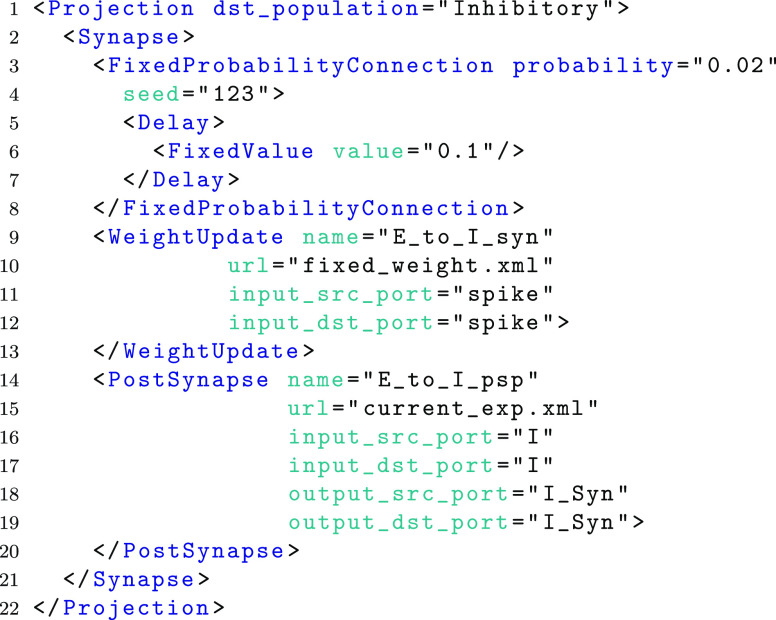


The *WeightUpdate* (line 9) and *PostSynapse* (line 14) element definitions follow the same property syntax as that of a neuron body. A *url* and *name* attribute must be specified however *size* is omitted as it is dynamically derived by either the connectivity pattern or the destination population size respectively.

Additional attributes within *WeightUpdate* and *PostSynapse* describe the dynamics connectivity of component ports to form a chain consisting of the pre-synaptic population, the weight update, the post-synapse and the post-synaptic neuron models. Within the *WeightUpdate* element the input source attribute (line 11) and input destination attribute (line 12) name the ports in which the pre-synaptic neuron (source) component connects to the weight update component (destination). Weight update input ports are typically linked by event ports which transmit instantaneous spike events. Within the *PostSynapse* element the input source attribute (line 16) and input destination attribute (line 17) name the ports in which the weight update component (source) connects to the post-synapse component (destination). Post-synapse input ports are typically linked by impulse event ports which, in the worked example, injects a current into the post-synaptic model following a spike event. The output source (line 18) and output destination (line 19) attributes within the *PostSynapse* element name the ports which link the post-synapse (source) to the post-synaptic population (destination). Analogue ports typically are used to emit current values from a post-synaptic model. An analogue reduce port within the post-synaptic neuron allows multiple post-synapse values to be summed. The *WeightUpdate* element has an additional, optional feedback source and destination port enabling information to be channelled from the post-synaptic neuron back to the weight update for learning (not required in the example). As with neuron bodies, both a weight update and post-synapse definition are used to specify properties (not shown). The full benchmark model including fully specified weight update and post-synapse properties is available from the tool-chain website.

### Extending the Network Layer

The use of high-level conceptual constructs such as populations and projections simplifies the processes of describing traditional point-based network descriptions. In particular the implied fan-out and fan-in of connectivity between neurons, weight update and the post-synapse items makes the high-level network description particularly compact and intuitive. Additionally the concept of a projection is shared by many neural simulators, simplifying the mapping processes during the code generation stage. Unfortunately, sole use of projection based connectivity is not suitable for describing communication other than chemical synapses (e.g. gap junctions and neuro-modulators). Consequently the ‘high-level’ object model is extended to form a ‘low-level’ object model (also shown in Fig. 5) which allows the direct connection of components via *Inputs* and *Groups* of component instances which do not specifically represent neuron bodies. The implications for simulators needing to support the ‘low’ level schema is that a generic (communication other than chemical synapses) method of connecting components must be available.

Within the low level network format, a *Group* object inherits *AbstractComponentInstance* and represents a set of component instances with a fixed size. Functionally a group is equivalent to a population with no projections, however the term group is used to distinguish between populations which do not represent neuron objects. The connection of a *Group* within a network is provided through the use of a generic *Input*. An *Input* uses a connectivity type to describe a remapping of an analogue, impulse or event value from a named *source* component. For worked examples of the low level network syntax, readers are directed to the Striatal modelling example which demonstrates gap junctions connected via inputs, available in full on the SpineML website.

### The Experimental Layer

The final phase of specifying a model is describing a simulation, to be conducted. The syntax of the experimental layer is similar to the SEDML experiment design language (Waltemath et al. 2011) but adds essential support for experiment inputs. It consists of details including the network model to be simulated, the period of simulation and the numerical integration scheme to be used. In addition to specifying model outputs, the experimental layer also supports the definition of model inputs, both of which interact directly with the dynamics of a model by logging or sending data to named component ports (shown in Fig. 2).

The following example defines an experiment which indicates a single independent simulation. The *Model* element (line 4) specifies a single attribute which references a network layer model. The *Simulation* element (line 5) specifies the simulator, length of simulation time, simulation duration and integration method. In this example, forward Euler integration is used although Runge Kutta methods are also supported. No explicit inputs are required within this experiment file as spike activity within the model is self propagating. Outputs are recorded from the simulation in the form of a *LogOutput* which specifies a log name (log file name) a *target* network layer *ComponentInstance* name and a *port*. No distinction is made between event ports or analogue ports. Each supporting simulator will check the port type and log either event times or values accordingly. 
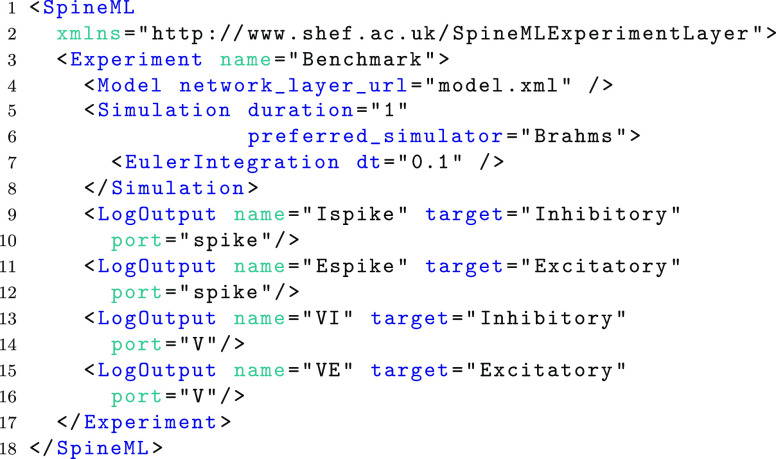


## Simulation Toolchain and Results

**Fig. 6:**
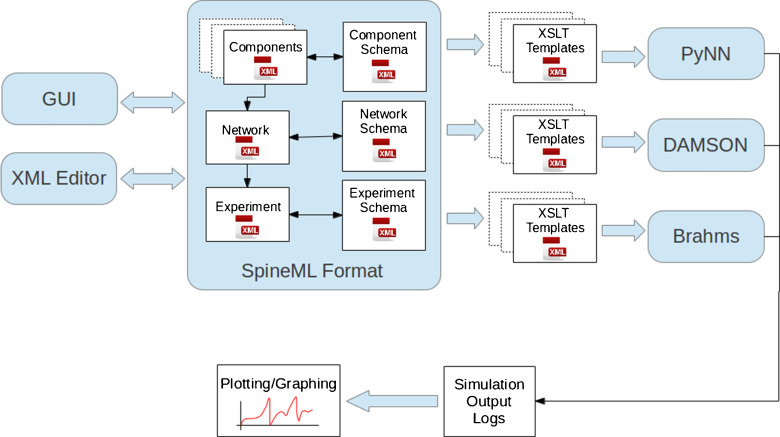
A tool-chain for simulation through code generation using the SpineML modelling syntax. The SpineML modelling syntax is composed of the Component, Network and Experiment XML description layers, where each is structured according to an XML Schema. Models can be generated manually using XML editors or using graphical user interface (GUI) tools. Translation of a model to any simulator is achieved by using a simulator specific set of XSLT templates to generate simulator code or native simulator model descriptions. Simulator code then logs results in a standardised format which can be used for plotting and analysis

To demonstrate the simulator independence of models completely described within SpineML, a tool-chain is proposed which uses eXtensible Stylesheet Language Transformations (XSLT) to generate native simulator code through transformation of SpineML model descriptions into plain text formats. Figure 6 shows the tool chain structure where each supported simulator uses a specific set of templates to generate simulation code. Each object modelling layer can be validated using an XML schema which defines the syntax and structure of each modelling layer using the XML language. Whilst XML models can be generated by hand, the use of a declarative common format allows independent tools to be generated for model design and creation using SpineML as a common storage format. Prototype graphical tools have been constructed for this purpose and will be the subject of future work.

Currently code generation templates have been developed for a reference simulator, BRAHMS (Mitchinson et al. 2010) (see section “BRAHMS”), a multi-processor multi-threaded event-driven form of C designed for emulating and compiling code for the SpiNNaker (Plana et al. 2007) hardware architecture, DAMSON (see section “DAMSON”) and a number of other simulators via PyNN (see section “PyNN”). Generating simulation code using the XSLT code generation tool-chain (as shown in Fig. 6) requires an XSLT 1.0 compliant processor. For example, using XSLTProc, simulation code for the PyNN simulator can be generated using the following command line argument. 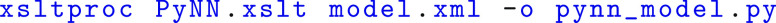


**Fig. 7:**
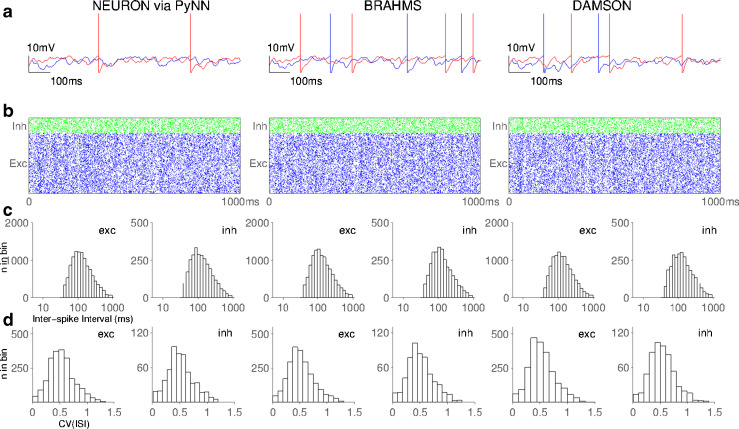
Results of running the worked example described using the SpineML format in three different supported simulators. The spike times and membrane potentials of two neurons were logged in NEURON via PyNN, BRAHMS and the DAMSON emulator and subsequently post processed to produce the resulting graphs **a** through to **c** which are consistent with results reported in (Davison et al. 2009; Nordlie et al. 2009) and also with results reported by a model designed natively in PyNN. **a** Shows the membrane potential for two excitatory neurons. Traces diverge after a short period of time due to differences in numerical integration between simulators which is compounded by the complexity of network activity. **b** Shows the spike raster plot of excitatory (*blue*) and inhibitory (*green*) neurons. **c** Shows the distribution of pooled inter spike intervals (ISIs) for excitatory (exc) and inhibitory (inh) neurons. **d** Shows the distribution of the coefficient of variation of the ISI over the populations of neurons

The generated simulation code can then be compiled or loaded using the simulator specific execution technique. Figure 7 shows a comparison of the results from running the above benchmark model in all three supported simulators; NEURON via PyNN, BRAHMS and the DAMSON emulator. In the case of PyNN, post-synaptic behaviour is intrinsic to the model of a neuron body. PyNN therefore requires a modified LIF neuron body component (which integrate post-synaptic behaviour as part of the neuron body). Each of the other supported simulators has been checked for consistency using the modified LIF component. A more detailed discussion of PyNN compatibility is given in section “System Implementation”.

### Reproducing a Model of the Striatum

**Fig. 8:**
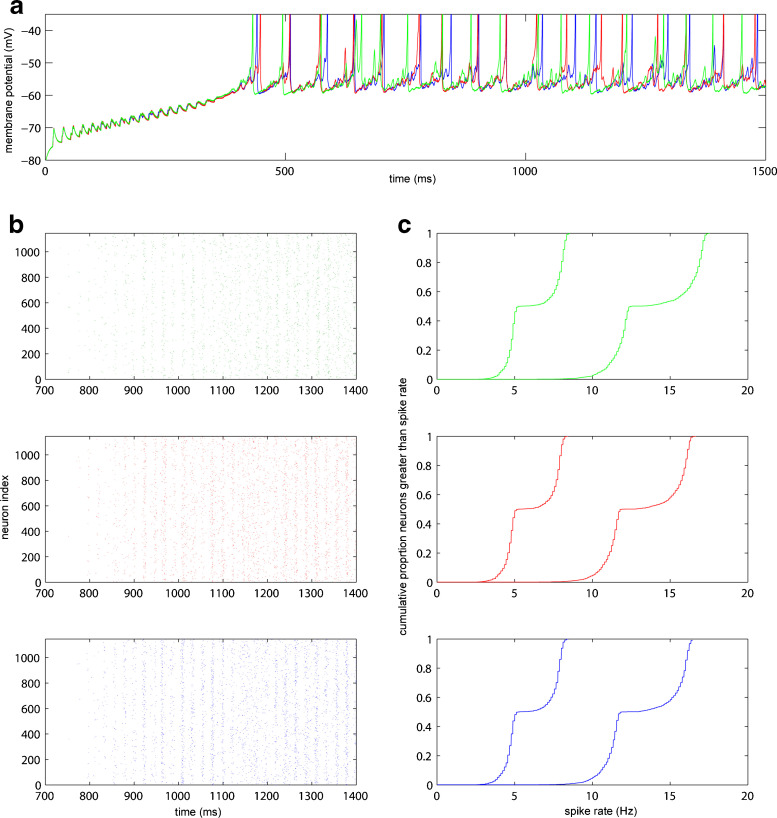
Statistical and Numerical comparison of a Striatal microcircuit model simulated in Native C++ and SpineML via BRAHMS and DAMSON. The Figure shows; **a** A numerical comparison of the membrane potential of a single Medium Spiny Neuron (MSN) over one and a half seconds of simulation in the C++ model (*Green*), BRAHMS (*Red*) and the DAMSON emulator (*Blue*). **b** Raster plot of MSN D1 neurons spike times over 700-1400ms of the simulation. Colours as for **a**. **c** Cumulative proportion of neurons (y-axis) with an average spike rate greater than the frequency (x-axis) for a simulation (at two input frequencies, *F* _*in*=4Hz and 5Hz) over a duration of 10 seconds (discarding the first second as this is when the model is reaching equilibrium). Colours as for **a**

The Striatal model was originally composed of large sections of C++ code with some smaller sections of MATLAB scripting (for generating connectivity and parameters). It proved straightforward to fully represent the model using SpineML with the low level network format. Generic components are used to form models of gap junctions and generic inputs are used in order to connect these to neural cell models. Owing to the use of the low level network layer, it is not possible to generate code for simulation in PyNN, therefore the results from simulations using BRAHMS and DAMSON code generation are reported. In order to test the veracity of the model against the original Matlab and C code version the statistical and numerical performances are compared with both SpineML simulations, as shown in Fig. 8. The results demonstrate that the statistical and numerical outputs of the model from the original C++ are reproduced by code generated from SpineML in both BRAHMS and the DAMSON emulator.

## System Implementation

The flexibility of the SpineML Syntax and the robustness of the XSLT code generation tool-chain are demonstrated by implementing three complete sets of templates for each of the three supported simulator back ends. Each set of translation templates varies from generation of a simulator configuration files (BRAHMS), to the generation of simulator API code (PyNN and BRAHMS), through to the generation of low-level C like code (DAMSON) for simulation on neuromorphic hardware. Because the range of code generation outputs for each of the supported simulators is different, specific code templates are required for each simulator (without shared templates). The advantage of unique simulator templates is that optimisations for each simulator level ensure optimal performance. Adding support for a new simulator requires that a suitable XSLT template (or set of templates) is constructed to map the dynamics or the component layer models, the structure of the network layer and the simulation details of the experimental layer. Simulators which have a close correlation with the SpineML object model (such as PyNN) require simpler code generation templates. The range (in terms of simulator architecture) of currently supported simulators also provides a good starting point for adding new simulator support.

The following sub-sections describe the design of the SpineML Schemas with particular emphasis on the extension mechanisms used to differentiate between low and high-level network descriptions and to add new network layer functionality. The XSLT code generation strategy is also presented with examples, followed by a description of the implementation of code generation templates, including any limitations, of each of the three supported simulators.

### Flexible XML Schemas

Various techniques can be used to design XML Schemas which range in their simplicity and flexibility. The simplest of these is often referred to as a ‘Russian Doll’ design and comprises a highly nested set of elements where only the root element has a global scope. Whilst very compact, this design technique is highly self-contained and changes made to types within the narrow local scope are not propagated to other Schemas or global definitions. The ridged structure offers similar functionality to Document Type Definition (DTD) validation but is unable to take advantage of type reuse. In contrast, the ‘Flat’ model (or sometimes known as the Salami Slice design) is highly reusable and consists of entirely globally defined elements which may be referenced by other element definitions within a Schema (or within Schemas which import it). The Flat model offers the ability to exploit substitution groups which offer similar functionality to object oriented polymorphism. This capability is desirable as new extensions can be easily integrated without daisy chaining changes in a schema. The final schema design type, the ‘Complex Type’ model (or Venetian Blind design) consists of a hierarchy of globally defined complex types with a single root element. This schema design method allows type extensions or restrictions offering functionality similar to object oriented inheritance. Consequently, this design type is used within NineML, NeuroML and LEMS and provides a sound method for designing schemas to describe hierarchies of increasingly complex neuron types. Its limiting factor is that it does not support the use of substitution. Therefore, new additions require the original schema to be changed.

With respect to SpineML there are a number of points where future extensions are highly desirable. Connectivity types are one such example where new connection primitives, such as distance-based connectivity or CSA (Djurfeldt 2012), may need to be added. In order to create a mechanism to allow the maximum degree of schema flexibility, a hybrid of the Flat and Complex Type schema methods has been used. This is the only method which affords the advantages of both inheritance and polymorphism. Within the Schemas, concrete element definitions are defined for each complex type at the global scope. These are referenced within other complex types and can therefore form the head of potential substitution groups where new types may be used. For example, the complex type (lines 1-3) and (abstract) global element definition (lines 5-7) for an *AbstractConnection* are shown below; 
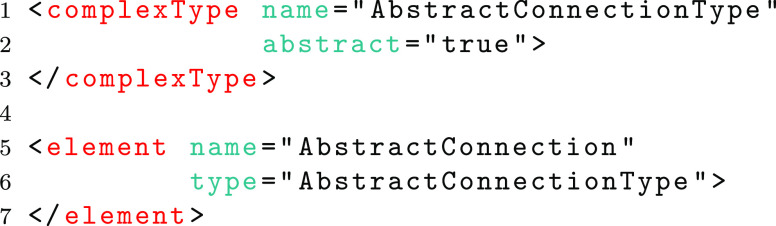


The global *AbstractConnection* element may be referenced within other type definitions. In the case of connectivity the *AbstractConnection* element is referenced within a *SynapseType* as follows. 
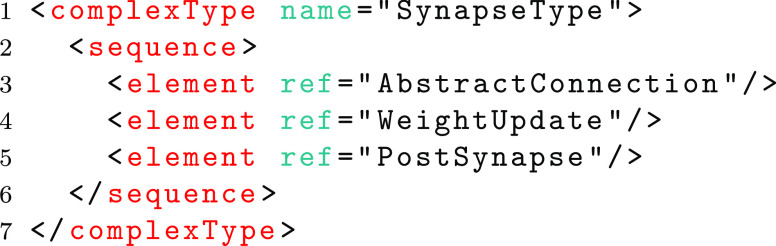


The inheritance mechanism of the complex type method then allows a new complex type to be defined as an extension to the *AbstractConnectionType*. In the example below, the definition of a type for one-to-one connectivity is shown (on lines 1-9). This type adds an element (Delay). A concrete element which references this new type (lines 11-14) can then specify that this element can be used in substitution with the *AbstractConnection* element. 
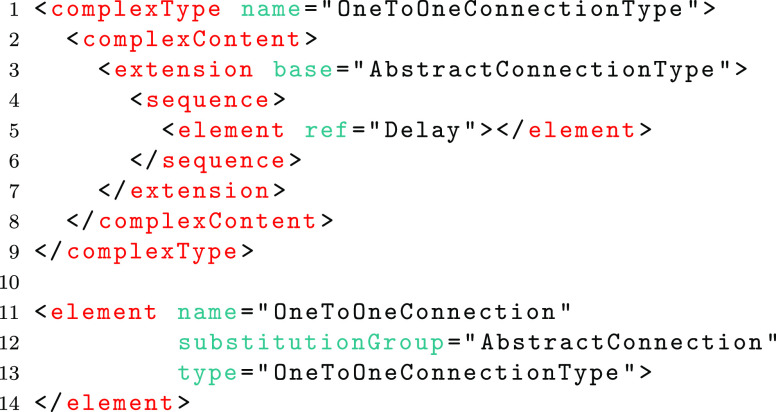


The above method provides an extension mechanism that allows a core schema to remain unchanged whilst new schemas are proposed to add new functionality such as connection types. This method is used to enable the addition of Groups with the low level schema and to ensure that the low level schema forms an extension of the high-level schema, adding new functionality in the form of inputs for all types of component instances (e.g. Neuron, WeightUpdate and PostSynapse).

### XSLT Code Generation

The mapping of a SpineML model to a simulation engine requires translation of the model using code generation (Goodman 2010). Code generation for SpineML has been provided through the use of XSLT templates. XSLT is used widely in the translation of XML documents to other HTML or other XML document formats on the web. Nevertheless, there is no restriction on the type of file that can be generated from an XSLT template and it is hence suitable for the generation of native simulator source code. XSLT standards are well defined and a number of compliant processors are available which can be used to generate identical output (e.g. XSLTProc, Saxon, Xalan and Visual Studio). In all cases, an XSLT processor works by recursively querying XML nodes through XPath expressions and applying a template to process the content of each node. In the case of generating simulator code, a model is processed by querying experiment, network layer and component layer documents recursively using the branching and control elements of XSLT to generate plain text.

XSLT affords several advantages over designing a custom template system. For example, XSLT can be used as a full functional programming language allowing complex data queries and output functions to be constructed. As XSLT is based upon XML technology, it is also possible to import schemas for each of the three SpineML modelling layers to ensure that models are validated before they are processed. Furthermore the XSLT templates themselves can be validated against the W3C XSLT Schemas. The following example (taken from the PyNN code template) shows how a simple XSLT query can be used to loop through all populations within a network layer to output Python code consisting of a population size variable using the neuron name. Complete documentation on the complete XSLT syntax is available on the w3schools website (http://www.w3schools.com/xsl/). 
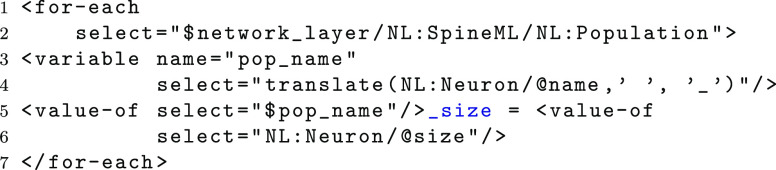


The *for*-*each* statement loops through each element which matches the *select* attributes XPath expression. The XSLT variable $*network*_*layer* refers to a network layer document. Within the *for*-*each* loop a new variable *pop*_*name* is constructed from the populations neuron *name* attribute. The function *translate* replaces any blank space character with an underline to comply with Python syntax rules. The *value*-*of * element then outputs the text value of the population name followed by the value of the neurons size attribute. The resulting output from the working example presented within section “Object Model and Examples”, is shown below; 
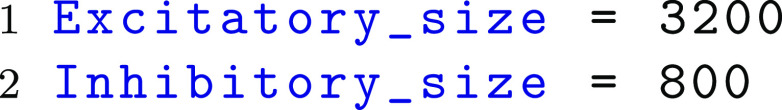


### BRAHMS

BRAHMS is described as middleware for integrated systems computation which has roots in the domain of biological modelling. Internally it contains a modular based execution framework (MEF) which links a number of software components with varying levels of abstraction. It has been used extensively for the simulation of spiking neural systems (Sullivan et al. 2012; Fox et al. 2009). As a result of its modular design, there is a straightforward conceptual mapping from the SpineML modelling syntax. BRAHMS supports both the high and low-level network format without any restrictions and serves as a reference simulator.

### PyNN

**Fig. 9:**
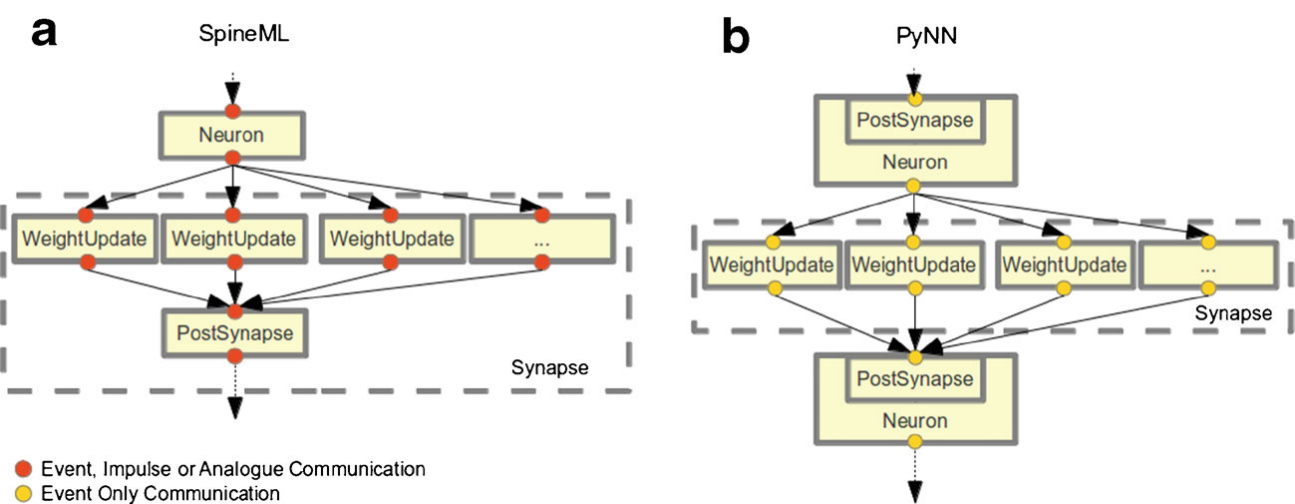
A comparison on the SpineML and PyNN synapse and post-synaptic response models. The Figure shows; **a** The SpineML synapse encapsulates separate synaptic weight update and post synapse models with a clear separation between the synapse and neuron body model. Typically a neuron will connect to a weight update via event-based communication, weight updates will connect to a post synapse using impulse communication and the post synapse will connect to a projected neuron via analogue communication. There is however no restriction imposing this and any form of event/impulse/analogue communication can occur between models. **b** PyNN combines the model of post-synaptic response with the model of a post-synaptic neuron. The synapse model (encapsulating only the weight update) is responsible for modelling the synaptic delay, weight and any dynamic behaviour of the synaptic weight. Within PyNN only event-based communication between a neuron (including the post-synaptic model) and a weight update is supported however weight updates may have non event-based internal dynamics i.e. short or long term plasticity

The PyNN simulator utilises a Python representation of a neuronal network model which is used to build a set of internal data structures. This internal model can then be simulated using a range of common simulators by passing the model structure through simulator specific APIs (usually with Python bindings). In order to translate a SpineML model into PyNN a translation template maps a model description into a single Python script which imports the respective PyNN modules. Support for the ‘low-level’ network layer is not possible within PyNN as only synaptic connectivity is supported through projections. The PyNN ‘synapse’, shown in Fig. 9, differs from that of SpineML. The encapsulation of the post-synaptic response model within a neural cell model prevents multiple projections to a single population which express different post-synaptic responses. In contrast, SpineML provides a separation of neuron and post-synaptic response model to allow any number of differing post-synaptic responses to a single population. To retain compatibility with PyNN, any neuron body components referenced within the network layer must match the implied dynamics of one of the standard PyNN library neuron model types, therefore incorporating the post-synaptic response. A ‘PyNN_PSP.xml’ model can then be used as a ‘pass through component’ which, rather than modelling the post-synaptic response, performs only a simple summation of any synaptic values, which are immediately passed through to a post-synaptic neuron model. Alpha based post-synaptic dynamics within a neuron model are calculated according to the methods described in Srinivasan and Chiel (1993) which avoids both the computational and storage overhead of computing synaptic conductance for each weight update in the network. Currently, only a fixed-weight update is implemented although support for learning can be easy integrated in the future by creating appropriate weight update models. A weight update feedback attribute provides a port for communication from post-synaptic neuron models.

Translation of a PyNN model to SpineML has not yet been implemented. However PyNN stores a model description by building an internal ‘flat’ model representation within memory. Any procedural techniques used within Python to generate properties or connections within PyNN can be converted to explicit lists and exported to the SpineML network layer format.

### DAMSON

Given the scale and power efficiency of the SpiNNaker hardware system, aimed at a million ARM processing cores supporting up to a billion real-time neurons, translation of SpineML to a suitable SpiNNaker format is highly desirable. The DAMSON^3^ language is an event-driven version of C with both SpiNNaker hardware support and functional emulation. Templates for generating DAMSON code map individual populations to SpiNNaker processors and create functions for handling simulation events (either spike based or analogue) which are routed between SpiNNaker cores as broadcast messages. In the case of large population sizes, an additional splitting stage (beyond the scope of this paper) can be used to aid parallelisation by evenly distributing the model across processing cores. Owing to the limited functionality of SpiNNaker ARM processing cores, all arithmetic within DAMSON is performed using an integer fixed-point format. The simulation results within this paper and from previous work (Sharp et al. 2012) demonstrate that this resolution is sufficiently accurate if scaling is handled carefully. The flexibility of the DAMSON language allows full implementation of both the high and low-level network layer formats with a few restrictions relating to weight update dynamics which are imposed by the requirement of real-time simulation. The first of these restrictions is that weight update components must (currently) be entirely event-based to reduce computational load. This constraint requires that the weight update component model communicates only via event or impulse ports and does not contain any time derivative within any of its regimes. For the same reason weight updates are not able to have generic inputs. Although it is possible to circumvent both restrictions by splitting a projections weight update and post-synapse into separate generic components (which will be allocated to different cores) future work will examine more effective ways of reducing the computational load per processing core in order to handle non event-based weight updates.

## Discussion

In this paper, SpineML, a declarative XML syntax extending the NineML format has been presented. The proposed extensions to NineML allow complete specification of complex biologically constrained point neuron network models, demonstrated in this paper through the specification of a model of the striatum. A code generation tool chain demonstrates the completeness of the syntax and its suitability for code generation with respect to point neuron networks of varying complexity.

Flexibility of modelling, in order to allow a wide range of biological phenomena to be represented, has been addressed within the SpineML network layer. The modular design provides a separation of the post-synaptic model and the model of a neural cell. The extension point mechanism allows a distinction between models containing only projection-based connectivity (high-level) and models which contain additional non-neural components and communication through mechanisms other than chemical synapses. The separation of the post-synaptic response is an important factor in reproducing biologically constrained models. For example, within the Striatal model, different post-synaptic dynamics are required for the NMDA component of the glutamatergic synapses of Medium Spiny Neurons (MSNs) compared to the GABAergic synapse and AMPA component which require a term to reproduce the effect of a blocking Magnesium current. Similarly, the post-synaptic currents have differing effects on AMPA and NMDA receptors in D2 MSNs and D1 MSNs respectively.

Both the PyNN API and NeuroML provide an alternative approach to model standardisation for simulator independent point neuron modelling. PyNN uses the high-level Python programming language to unify a range of simulators which have Python interfaces (Hines and Carnevale 1997; Eppler et al. 2009; Goodman and Brette 2008). The drive towards Python as a common language for simulators is understandable. As a high-level language it is open source, has a simple yet powerful syntax and supports extensive libraries for scientific computing data analysis and visualisation. NeuroML v1.0 is a declarative XML based description language for model specification and exchange with a focus on morphological neural systems. NeuroML v2.0^4^ has extended its remit to also include the specification of point neuron networks. It has been developed alongside the Low Entropy Model Specification (LEMS) for specifying generic models of dynamical systems. The NeuroML v2.0 schemas provide a hierarchical structure of cell and synapse models which make reference to LEMS components (called ComponentTypes). A NeuroML v2.0 model description can therefore be used to either map a cell description to native simulator parameters or be used in conjunction with LEMS for code generation.

A comparison of SpineML can be made with PyNN and NeuroML both with respect to specification of neural components and networks. In terms of component specification, the SpineML component layer makes relatively few changes to the NineML abstraction layer; the two are largely compatible and have some degree of overlap with LEMS (and hence NeuroML). SpineML differs from PyNN in that there is a focus on more flexible (biologically constrained) model specification (e.g. allowing specification of new neuron, learning or post-synaptic response models), rather than on extensive simulator support. As a result, PyNN is unable to provide the functionality for describing new neural components in a simulator independent fashion. Some initial attempt at integrating LEMS and the NineML abstraction layer with PyNN have however been made. The integration relies on non-standard PyNN neurons being translated to simulator specific implementations through code generation with only the network described by PyNN. The NeuroML v2.0 schema defines support for point neurons with a strong overlap with standard PyNN neuron types. Both PyNN and NeuroML v2.0 descriptions of PyNN standard cell types utilise a combined post synapse and neuron cell models (the disadvantages to this approach have been covered in section “PyNN”).

Both SpineML and NeuroML v2.0 present a large degree of flexibility in that LEMS can be used to describe new dynamics. Where NeuroML v2.0 and SpineML differ with respect to network specification in that a new LEMS model requires the additional specification of a compatible schema (extending one of the core NeuroML v2.0 model types) in order to provide XML validation of a parametrised (LEMS) model description. Within NeuroML v2.0 a parametrised model description can then be referenced by a population object with the advantage that the parametrised model may be reused by multiple populations. In contrast, SpineML does not require any additional schema specifications to allow XML validation for paramaterisation of new ‘component’ model. Neuron, synapse, post synapse (and groups) are instantiated directly within a network layer model description by referencing a component layer object directly. The underlying schema remains unchanged, simplifying the XSLT code generation process. Although SpineML is not currently able to reuse parametrised components, the decision to omit this functionality is based on the need to be able to visually represent the networks of instantiated components within graphical editing tools. How parametrised but unused components would be represented within a conventional visual design tool is not immediately clear but will be considered in future work. PyNN’s lack of support for connectivity through mechanisms other than chemical synapses within a network description has already been documented. Aside from differences in the specification of synapses, SpineML and PyNN differ in that PyNN utilises a procedural programming language. Although Python code can be written in a declarative way, the procedural nature of the Python language can make it more difficult to infer the overall structure of a model and discovering the value of a modelling parameter can often be less intuitive than in a purely declarative specification.

Considering model storage, XML is advantageous in terms of readability and portability but can perform poorly in file size. Models containing large explicit lists of properties of connectivity can easily become unwieldy due to the mark up notation used. XML tags contain large data redundancy although compression techniques work well in reducing file sizes. Alternatively, binary file formats for explicit lists will be added to the high-level network schema format at a later date. Additional distance based connectivity primitives would also help to alleviate file size for models such as the Striatal example where complex distance based connectivity functions (Humphries et al. 2010) are currently described as explicit lists, contributing to over 99 % of the total file size.

Performance of the XSLT processor in translating a model to native simulator format is dependent on the simulator specific templates. XSLT stores both a model and the template entirely in memory (within a DOM tree) ensuring that XPath queries are fast, restricting models to those which fit entirely in memory. Although the benchmark model presented within this paper has been scaled to gigabyte network layer model sizes and has been used for code generation, beyond this scale, the use of streaming templates may be necessary to reduce memory usage. Alternatively a combination of XSLT to generate native simulator ‘programs’ with a more optimised streaming code generation method for native simulator ‘data’ is entirely feasible.

The main focus for future work will be in advancing the development of graphical user tools for declarative model specification. Current prototype tools require the addition of a layout layer for saving layout of neurons and populations within 3D space for use in both generating complex spatially-based connectivity patterns and visualisation. Integration between SpineML and NineML will be pursued via community driven discussions. Translation to a range of other simulator formats including NEST and NEURON which each offer support for custom components will also be considered as will support for GPU based simulation.

## Information Sharing Statement

The model layer schemas and example models presented within this paper (namely the benchmark model and Striatal model) are available in complete form from the SpineML website (http://bimpa.group.shef.ac.uk/SpineML). Additional documentation and additional simulator configurations are also available to aid users in reproducing the experiments and results presented.

## Footnotes

1
http://www.neuroml.org/lems/

2
http://www.neuroml.org/neuroml2.php

3
http://damson.sites.sheffield.ac.uk/

4
http://www.neuroml.org/neuroml2.php
